# Interspecific effects of invasive wild pigs (*Sus scrofa*) on native nine-banded armadillos (*Dasypus novemcinctus*)

**DOI:** 10.1093/jmammal/gyaf023

**Published:** 2025-04-21

**Authors:** Matthew S Broadway, Holly M Todaro, Molly M Koeck, Courtney N Dotterweich, Sarah A Cain, Lindsey Buehler, M Colter Chitwood, Robert C Lonsinger

**Affiliations:** Department of Natural Resource Ecology and Management, Oklahoma State University, 008 Agriculture Hall, Stillwater, OK 74078, United States; Department of Natural Resource Ecology and Management, Oklahoma State University, 008 Agriculture Hall, Stillwater, OK 74078, United States; Department of Natural Resource Ecology and Management, Oklahoma State University, 008 Agriculture Hall, Stillwater, OK 74078, United States; Department of Natural Resource Ecology and Management, Oklahoma State University, 008 Agriculture Hall, Stillwater, OK 74078, United States; Department of Natural Resource Ecology and Management, Oklahoma State University, 008 Agriculture Hall, Stillwater, OK 74078, United States; Department of Natural Resource Ecology and Management, Oklahoma State University, 008 Agriculture Hall, Stillwater, OK 74078, United States; Department of Natural Resource Ecology and Management, Oklahoma State University, 008 Agriculture Hall, Stillwater, OK 74078, United States; U.S. Geological Survey, Oklahoma Cooperative Fish and Wildlife Research Unit, Oklahoma State University, 007 Agriculture Hall, Stillwater, OK 74078, United States

**Keywords:** Camera trap, *Dasypus novemcinctus*, diel activity, Eurasian Wild Pig, invasion, Nine-banded Armadillo, occupancy, species interactions, *Sus scrofa*

## Abstract

Biological invasions pose significant risks to ecosystems and native species. Wild pigs (*Sus scrofa*) are a highly detrimental invasive species in North America, directly and indirectly affecting native species. Co-occurrence of wild pigs and native species may lead to interspecific interactions that alter ecological communities. Accordingly, we investigated spatial and temporal factors influencing detection and occupancy of Eurasian Wild Pig and Nine-banded Armadillo (*Dasypus novemcinctus*) before examining interspecific effects. We analyzed camera-trap data collected from August to September 2021 using a hierarchical modeling framework to estimate detection and occupancy of both species individually (single-species analyses) and concurrently (conditional co-occurrence analyses). We observed higher Wild Pig detection rates and space use in late summer and in areas with greater riparian cover, respectively. Armadillo detection increased linearly throughout our sampling season and in response to precipitation. Moreover, armadillo detection was 3.5 to 5.1× higher at sites used by wild pigs, regardless of whether wild pigs were detected during a survey period. Occupancy of armadillo was best explained by a quadratic trend in site elevation but did not depend on the presence of wild pigs. Our results indicate that wild pigs may influence armadillo detection (or site-use intensity), but not occupancy, therefore revealing nuanced interspecific interactions. Between species, we observed high overlap in diel activity but significantly different activity peaks, with armadillos being strictly nocturnal and wild pigs being crepuscular but with more cathemeral activity, suggesting that fine-scale temporal partitioning may have occurred. Our results provide insights into the influence of a large-bodied and destructive invasive species (Wild Pig) on a smaller, ecologically important native species (Nine-banded Armadillo).

## Teaser

Invasive species may modify native species occurrence and behavior through indirect and direct mechanisms. We studied the influence of wild pigs on Nine-banded Armadillo providing evidence that wild pigs modify armadillo behavior. Armadillo activity was 3.5 to 5.1× higher in areas used by wild pigs.

Species invasions pose significant threats to global biodiversity (Levine 2008; Simberloff et al. 2013) by modifying ecosystem structure and function through indirect and direct mechanisms (Stachowicz 2001; Levine 2008; Peltzer et al. 2010; Van Klink et al. 2020). Invasive species also facilitate novel interactions that result in positive or negative effects on native species (Daly et al. 2023), with the direction and strength shaped by factors such as existing ecological processes, trophic level, biodiversity, behavioral plasticity, or niche overlap (Ruscoe et al. 2006; Altieri et al. 2010; Wright et al. 2010). For example, invasive species occupying higher trophic levels (i.e., predators) typically exert strong direct negative effects on native prey (Medina et al. 2011; Doherty et al. 2016). Contrastingly, invasive species may benefit native species by modifying interactions that determine animal distributions in space and time (Svenning et al. 2014; terHorst et al. 2015). These effects can arise through mechanisms such as altered predator-prey dynamics, reduced interspecific competition, or altered habitat conditions (Williamson 1996; Rodriguez 2006; Svenning et al. 2014; terHorst et al. 2015). In some cases, invasive species may even facilitate the spread of others in cascading effects through ecosystems (Simberloff and Von Holle 1999; Rodriguez 2006; Davis et al. 2010; Svenning et al. 2014; Aslan et al. 2015).

**Fig. 1.**
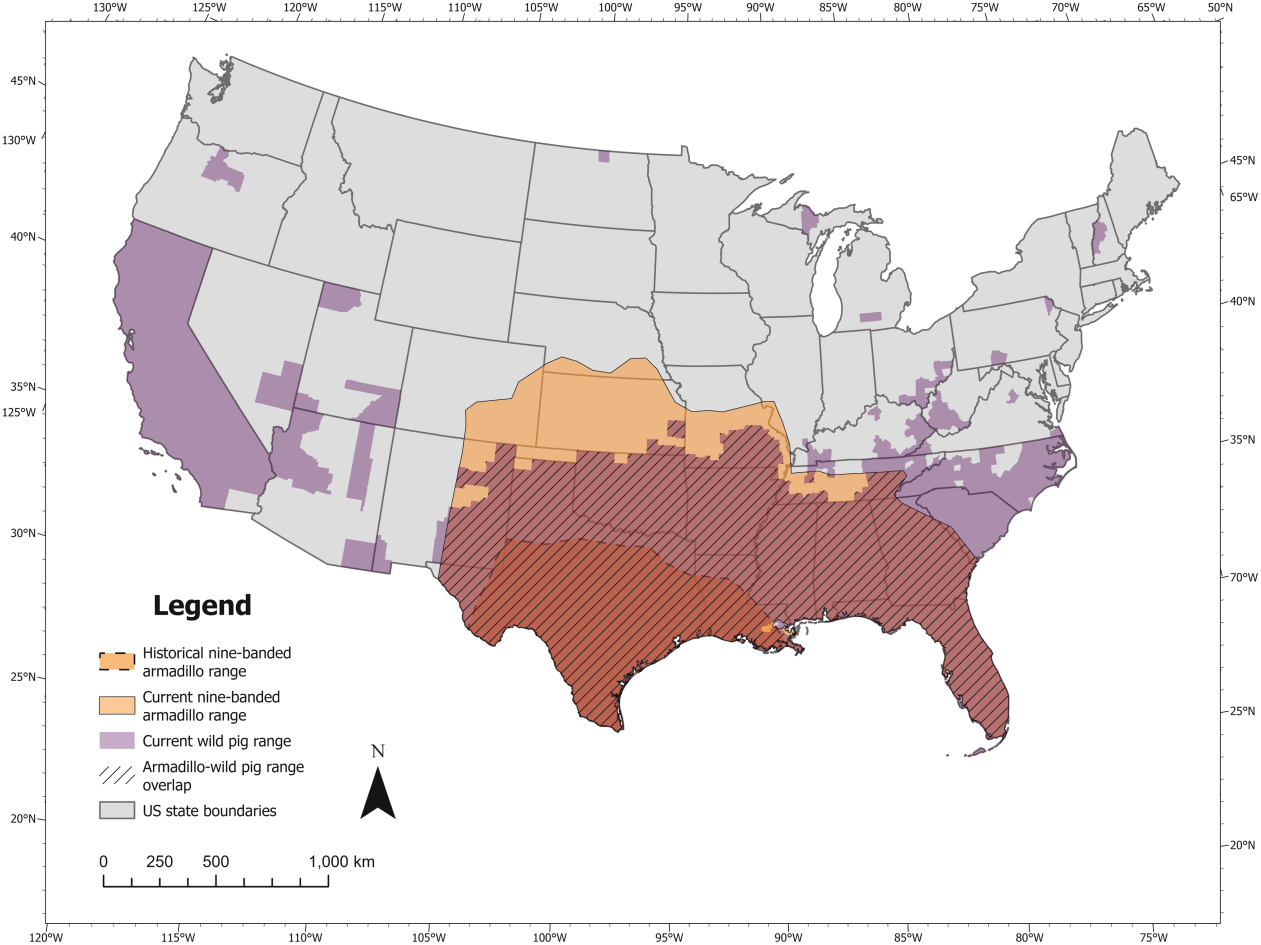
Range map illustrating the distribution of wild pigs (*Sus scrofa*) and the historical and current distributions of nine-banded armadillos (*Dasypus novemcinctus*) within the contiguous United States, highlighting areas of sympatry. Armadillo historical and current ranges were created using publicly available spatial data provided by NatureServe and Tennessee Wildlife Resources Agency, and the IUCN (2014), respectively. Wild pig ranges were created using the US Geological Survey (USGS)—Gap Analysis Project (GAP 2018).

Invasive Eurasian wild pigs (*Sus scrofa*; hereafter, wild pigs) and native nine-banded armadillos (*Dasypus novemcinctus*; hereafter, armadillos) represent a unique pairing to evaluate the effects of concomitant biological invasions. Wild pigs and armadillos are sympatric throughout much of their ranges in the United States (Fig. 1) and both have experienced recent range expansions (Loughry et al. 2013; Bevins et al. 2014; McClure et al. 2018; DeGregorio et al. 2021). Wild pigs are ecologically destructive (e.g., through their rooting behavior) and their direct negative effects on plant and animal communities are well documented (Massei and Genov 2004; Barrios-Garcia and Ballari 2012; Risch et al. 2021; McDonough et al. 2022). In contrast, armadillos provide significant ecosystem services (e.g., bioturbation, seed dispersal, and pest control; Rodrigues et al. 2020; DeGregorio et al. 2022; Lamb et al. 2023) but are commonly considered a nuisance due to damage in suburban environments (Ober et al. 2011) and risks posed to human health (i.e., as a carrier of *Mycobacterium leprae*, the causative pathogen of leprosy; Loughry et al. 2013).

Wild pigs and armadillos demonstrate spatial overlap, suggesting potential interspecific interactions (McDonough et al. 2000; Gammons et al. 2009a). Additionally, overlapping temporal activity patterns may further increase the probability of interactions between wild pigs and armadillos (Saldo et al. 2023). Wild pigs forage by uprooting soil and consuming plants, vertebrates, and invertebrates (Marshall et al. 2020; Canright et al. 2023). Destructive rooting behavior by wild pigs may influence habitat conditions and foraging strategies of armadillos (Sikes et al. 1990; White 1992; Barrios-Garcia and Ballari 2012), thus altering spatiotemporal patterns of armadillo occurrence. Identifying interspecific interactions between wild pigs and armadillos may reveal a mechanism by which invading species impose community-level effects on local flora and fauna.

Our goal was to investigate how space use and detection of native armadillos were influenced by invasive wild pigs while accounting for environmental effects. We accomplished this by first examining the influence of environmental factors on the space use and detection of each species using single-species occupancy models (MacKenzie et al. 2002). Thereafter, we evaluated the influence of wild pigs on armadillo space use and detection with conditional 2-species occupancy models (Richmond et al. 2010). We used occupancy analyses to explicitly account for factors affecting species-specific detection in both single- and 2-species analyses. Additionally, we assessed the degree of average within-day temporal overlap between wild pigs and armadillos with activity curve analyses (Ridout and Linkie 2009). For each species, we tested a priori hypotheses regarding the effects of environmental factors on patterns of space use and detection for wild pigs and armadillos. We hypothesized that wild pig space use would be influenced by elevation, deciduous and riparian land cover, and stream proximity. We also hypothesized that wild pig detection would vary throughout the sampling season (e.g., due to changing mast availability) and by weather conditions (i.e., temperature and precipitation). We hypothesized that armadillo occupancy would be influenced by elevation, proportion of key land-cover types (i.e., deciduous forest, riparian, and shrub cover), and proximity to streams. We also hypothesized that armadillo detection would be influenced by temperature and precipitation. Following single-species analyses, we considered whether wild pig presence, detection, and temporal activity precipitated interspecific interactions in space or time with armadillos. We hypothesized that armadillo space use and detection would be influenced by wild pigs, consistent with patterns observed by Saldo et al. (2023) during spring. Finally, although nocturnality may vary as a function of environmental conditions (Burton et al. 2024), we predicted that both species would demonstrate nocturnal diel activity patterns with a high degree of temporal overlap (Saldo et al. 2023).

## Methods

### Study area.

We collected camera-trap data from 2 study areas—the James Collins Wildlife Management Area (35.0421° N, 95.4785° W; 8,500 ha) and the Sans Bois Wildlife Management Area (35.1109° N, 94.9675° W; 3,000 ha)—within the Arkansas Valley ecoregion in southeast Oklahoma. Both study areas encompassed a portion of the Sans Bois Mountains within the broader Ouachita Mountains ecoregion (EPA 2013), with elevations ranging from 180 to 500 m. The study areas were characterized by dense forest cover and rugged terrain. Dominant land-cover types included mixed forest (47%), evergreen forest (30%), and deciduous forest (15%). Mixed forests were associated with low elevations where riparian and wetland areas occur, but xeric conditions occurred at higher elevations owing to slope and well-drained soils. The eastern portion of the James Collins Wildlife Management Area was dominated by Shortleaf Pine (*Pinus echinata*) and Loblolly Pine (*P. taeda*) on ridges, whereas the western portion was primarily oak (*Quercus* spp.) and hickory (*Carya* spp.) forests interspersed with native grasslands. Sans Bois Wildlife Management Area was characterized by oak-pine-hickory forests on steep slopes, with flatter areas containing native grasslands and wetland areas at lower elevations.

### Camera data.

We deployed 50 Reconyx Hyperfire 2 Professional cameras within each study area (100 total) during August to September 2021 (Koeck 2023). We selected camera deployment sites using generalized random tessellation stratified sampling (Stevens and Olsen 2004), which generated spatially balanced random points, ensuring adequate representation of spatial heterogeneity. We outfitted cameras with 32-gigabyte secure digital cards and programmed cameras to take motion-triggered photographs in bursts of 3, with no delay between triggers. Cameras were active and functioning for the full diel period to collect diurnal and nocturnal detections. We positioned cameras <1 m above ground, aimed cameras parallel to the ground (and across slopes, as needed), and directed them northward (when feasible) to reduce sunlight on lenses. We did not use attractants and left cameras undisturbed for the entire deployment period. Upon recovery, we used Timelapse 2 (Greenberg 2019) to process images and identify species. For each target species, we coded detections within each survey period as a binary response variable indicating a detection (1) or nondetection (0). To limit model convergence issues and reduce potential bias associated with temporal autocorrelation in detections (Goldstein et al. 2024), we defined each survey as 1 wk (7 d), resulting in 8 surveys within the sampling season.

### Spatial data.

We identified environmental covariates predicted to influence space-use patterns of each species (Table 1). Despite being generalists, wild pigs have demonstrated selection for deciduous forests, riparian, and wetland areas (Bastille-Rousseau et al. 2020; Kramer 2021; Walters and Osborne 2022) and relative avoidance of shrub cover (Bastille-Rousseau et al. 2020). Higher proportions of riparian and deciduous forests surrounding sites may provide thermal refugia, travel corridors, or foraging opportunities, possibly increasing wild pig use. Similarly, armadillos are reportedly habitat generalists that may positively respond to hardwood forest availability (McDonough and Loughry 2005), avoid mature pine (Gammons et al. 2009b), and rely on water sources (Rodrigues and Chiarello 2018). Rodrigues and Chiarello (2018) observed a significant negative effect of distance to water on armadillo occupancy in South America. However, because water sources at our sites could be ephemeral and moist soils at low elevations were not always associated with classified water bodies, we used riparian land cover to include any moist soil area, rather than only those associated with water sources. We found no published results directly estimating the response of armadillos to shrub cover; however, previous observations of burrow locations under shrubs and nest construction (Taulman 1994; Bond et al. 2000) suggested that shrub cover may provide concealment from predators. Additionally, armadillos forage in ground litter and excavate soil for shallow macroinvertebrates (Redford 1985). Higher elevation soil substrates at our sites contained high rock fragment density and were presumably shallower, which may have limited food or burrow availability. We characterized land-cover covariates from the 2020 Landfire Existing Vegetation Type raster dataset with 30 m spatial resolution (LANDFIRE 2020) by aggregating vegetative communities into broader categories with 4 land-cover types of interest (i.e., conifer, deciduous, shrub, and riparian). Thereafter, we calculated the proportion of each land-cover type within a 200-m buffer around each camera, and the linear distance (m) of each camera site to nearest stream (using the same spatial data layer), with the “terra” package (Hijmans et al. 2023) in Program R version 4.3.1 (R Core Team 2023). We also extracted elevation (m) of camera sites from the US Geological Survey Digital Elevation Model data layer (U.S. Geological Survey 2018). We performed all spatial data curation, manipulation, and extraction in ArcGIS v. 10.3 (ESRI, Inc., Redlands, CA).

**Table 1.** Covariates used within single-season single-species occupancy models to explain detection (*p*) and occupancy (ψ) of wild pigs (*Sus scrofa*) and nine-banded armadillos (*Dasypus novemcinctus*; Armadillo) in southeastern Oklahoma, USA, during August to September (2021).

			Prediction^b^	
Parameter	Covariates^a^	Abbreviation	Wild pig	Armadillo
*p*	Time (trend)	*T*	+	+
	Time (weekly)	*T*_*week*_	+	+
	Time (biweekly)	*T*_*bi-week*_		
	Time Monthly	*T*_*month*_		
	Temperature	Temp	−	−
	Precipitation	Precip	−	+
				
ψ	%Shrub		−	+
	%Deciduous		+	+
	%Riparian	Rip	+	+
	Distance to Stream	Stream	−	−
	Elevation	−	−	−

^a^Covariates: Time = temporal variation in detection considered as a trend in detection or differences between weekly, biweekly, and monthly periods; Temperature = mean daily minimum temperature (°C); Precipitation = mean daily precipitation (mm); %Land cover = proportion of each land-cover type within a 200-m buffer; Distance to stream = distance (m) to nearest stream; Elevation = elevation (m) of site.

^b^Predicted positive (+) or negative (−) effect of each covariate for each species.

### Occupancy modeling.

We investigated spatial relationships between wild pigs and armadillos within an occupancy modeling framework (MacKenzie et al. 2018). We initially analyzed detection data within single-species single-season occupancy models to identify environmental factors influencing species-specific space use (ψ) and detection (*p*) patterns (MacKenzie et al. 2002). Occupancy analyses assume that sites are closed to changes in species-specific occupancy state over the sampling period (MacKenzie et al. 2002). Although research is limited on armadillo spatial ecology, we used 400 m buffers between camera sites to approximate their reported home range size (0.11 km^2^; Gammons et al. 2009b) and movement distances (<200 m; McDonough and Loughry 1997) to meet the closure assumption, and we randomly removed a camera from consideration when buffers overlapped. In contrast, our sites may not have met the closure assumption for wild pigs, which have large (mean = 15.5 km²; reviewed in Garza et al. 2018) and variable (e.g., seasonally and by sex; Mersinger and Silvy 2007) home range sizes. Violating the closure assumption of occupancy models can introduce positive biases in occupancy estimates (Rota et al. 2009), but occupancy can still be used to effectively characterize space-use patterns (Gould et al. 2019). Nonetheless, to identify evidence of model assumption violations, we assessed goodness-of-fit (GOF) for each single-species global model using 1,000 parametric bootstrap replicates on a *χ*^2^ statistic appropriate for binary data (implemented in “unmarked”; Fiske and Chandler 2011; Steenweg et al. 2016; Lonsinger et al. 2021). We used the results from our single-species models within a conditional 2-species occupancy model (hereafter, co-occurrence model) to assess the relative importance of environmental factors and wild pigs on armadillo space use and detection (Richmond et al. 2010).

### Detection modeling.

Failure to account for imperfect detection (i.e., detection probabilities <1) can lead to biased estimates of space use (MacKenzie et al. 2002); therefore, we identified environmental covariates expected to influence detection of each species (Table 1). Franckowiak et al. (2018) observed a decrease in wild pig movement with increasing temperature and increased precipitation in Texas. Armadillo activity has also been associated with weather variables (e.g., temperature, precipitation; McDonough and Loughry 1997; Gammons et al. 2009b; Saldo et al. 2023). To characterize temperature and precipitation variability during our sampling, we downloaded data from 2 environmental stations (each <30 km from both study areas) maintained by the Oklahoma Climatological Survey (https://mesonet.org/), which recorded weather data at 5-min intervals. We derived daily minimum temperature (°C; hereafter, temperature) and daily precipitation (mm) from each station before calculating mean weekly values for both variables across both stations and within each weekly survey period. Temporal variation in detection is common, and failure to account for temporal variation can lead to unmodeled heterogeneity in detection and bias estimates of occupancy (Sollmann 2024). For both species, we also considered temporal variation in detection and competing characterizations where detection varied linearly (i.e., a trend in detection over time), weekly (i.e., differences in detection among survey periods), biweekly (i.e., differences in detection by 2-wk intervals), or monthly (i.e., differences in detection during the first and second months of sampling).

Prior to developing candidate model structures for each species, we compared competing structures of a temporal model to explain stochasticity in detection for each species independently. Temporal variability in detection may be a consequence of behavioral changes in response to ecological conditions. Specifically, increasing availability of important food resources such as hard mast (Ditchkoff and Mayer 2009) could lead to increased movement, and therefore detection, of wild pigs. Accordingly, we compared variable temporal detection model structures as proxies for unmeasured ecological conditions. For each species, we assessed the relative fit of a model with a constant (intercept only) detection structure versus models with a time trend (*T*) or 1 of 3 discrete intervals—i.e., weekly (*T*_*week*_), biweekly (*T*_*bi-week*_), or monthly (*T*_*month*_) intervals—and retained the most-supported temporal detection structure for each species for later analyses. We tested for pairwise correlations between covariates using a Pearson correlation coefficient test and avoided using covariates correlated at |*r*| > 0.5 in the same model (Dormann et al. 2013). Subsequently, we modeled detection and occupancy for each species as a function of environmental covariates with single-species single-season occupancy models. For each species, we developed a candidate model set by considering all-possible-additive combinations of covariates within the models for detection and occupancy and all combinations of detection and occupancy models (Doherty et al. 2012). We conducted all occupancy modeling procedures in Program MARK version 10.3 (White and Burnham 1999), excluded models that failed to converge, and evaluated relative support for models with Akaike’s Information Criterion adjusted for small sample sizes (i.e., AIC_*c*_; Akaike 1973; Burnham and Anderson 1998). We assessed the influence of covariates on each species by considering the structure of the most-supported model in each analysis and assessing beta coefficients and associated 85% confidence intervals (i.e., CI; Arnold 2010). Additionally, we evaluated cumulative Akaike weights (i.e., variable weights) to assess relative importance of covariates while accounting for model uncertainty (Anderson and Burnham 2002; Arnold 2010).

### Co-occurrence modeling.

Following single-species occupancy models for wild pigs and armadillos, we tested 12 a priori hypotheses of interspecific relationships within a co-occurrence framework (Richmond et al. 2010; Table 2). Co-occurrence models included parameters for: (i) occupancy of wild pigs (i.e., the presumed dominant species; indicated with “A”; ψ^A^); (ii) occupancy of armadillos (i.e., the presumed subordinate species; indicated with “B”) in the presence (ψ^BA^) or absence (ψ^Ba^) of wild pigs; (iii) detection of wild pigs when armadillos were absent (*p*^A^) or present (*r*^A^); and (iv) detection of the armadillos when wild pigs were absent (*p*^B^), or when wild pigs were present and detected (*r*^BA^) or not detected (*r*^Ba^) during the survey. Due to the complexity of these models, we maintained constant structures for species-specific models by carrying forward the most-supported structures for environmental covariates on detection and occupancy of each species. Most initial co-occurrence models including environmental covariates failed to converge (see Results), so we ultimately excluded environmental covariates on occupancy within the co-occurrence framework but retained detection covariates to account for imperfect detection. The 12 candidate models described in Richmond et al. (2010) differed in how parameters were constrained to test for evidence of interspecific interactions. For example, comparing models where ψ^BA^*≠* ψ^Ba^ (i.e., an effect) to models where ψ^BA^ = ψ^Ba^ (i.e., no effect; ψ^B^) provides evidence for whether the occurrence of wild pig affects the occurrence of armadillo. We conducted co-occurrence analyses in Program MARK and evaluated relative support for models with AIC_*c*_ (White and Burnham 1999; Anderson and Burnham 2002).

**Table 2.** Descriptions of parameters estimated by the conditional 2-species occupancy model (adapted from Richmond et al. 2010).

Parameter	Description
ψ^A^	Species A occupancy probability
ψ^BA^	Species B occupancy probability, given species A is present
ψ^Ba^	Species B occupancy probability, given species A is absent
*p* ^A^	Detection probability of species A, given species B is absent
*p* ^B^	Detection probability of species B, given species A is absent
*r* ^A^	Detection probability of species A, given both species are present
*r* ^BA^	Detection of species B, given species A is present and detected
*r* ^Ba^	Detection of species B, given species A is present and not detected

### Temporal activity analysis.

We calculated the degree of overlap in diel activity between wild pigs and armadillos on a 24-hr scale. Time stamps from camera-trap images can be used to generate accurate estimates of animal activity patterns when effective sample sizes are met (Ridout and Linkie 2009; Rowcliffe et al. 2014; Lashley et al. 2018; Niedballa et al. 2019). We used package “overlap” (Meredith and Ridout 2014) in Program R v. 4.3.1 (R Core Team 2023) to estimate the coefficient of overlap between wild pig and armadillo activity for sample sizes > 75 (i.e., ∆_4_). We considered images of the same species separated by ≥30 min (i.e., latent period between detections) to be independent detections, which aligned with the independence threshold applied by previous studies for wild pigs and armadillos (Saldo et al. 2023) and produced activity results that were comparable to telemetry data for wild pigs (Wolfson et al. 2023). We subsequently derived associated 95% CIs from 1,000 smoothed bootstrapped samples and visualized overlapping kernel density estimates of activity patterns. Using package “circstats” in Program R v. 4.3.1 (R Core Team 2023), we performed Watson’s Two-Sample Test of Homogeneity, which is robust to violations of unimodally distributed data when sample sizes are large (Landler et al. 2021), to determine if species-specific activity times came from the same distribution; subsequently, if the distributions of species-specific activity times were heterogeneous, we performed a Watson’s Test of Uniformity of both species individually. For all circular statistical procedures, we set α = 0.05.

## Results

### Camera data.

We used 87 cameras in the analysis. Four cameras were stolen or damaged, and the remainder were removed because they did not meet the minimum distance required between cameras (i.e., 400 m). Across 87 camera-trap sites, we collected a total of 1,095,703 images and detected wild pigs at 21 (24%) sites and armadillos at 46 (53%) sites; we detected both species at 14 (16%) sites. For diel activity analyses, we identified 274 independent detections of armadillos and 637 independent detections of wild pigs. Evaluation of temporal detection structures revealed species-specific temporal variation in detection, with wild pig and armadillo detection being best explained using discrete monthly intervals (*T*_*month*_) and a linear trend (*T*), respectively (see below). We did not detect pairwise correlations between covariates with |*r*| > 0.5.

### Single-species occupancy and detection.

**Fig. 2.**
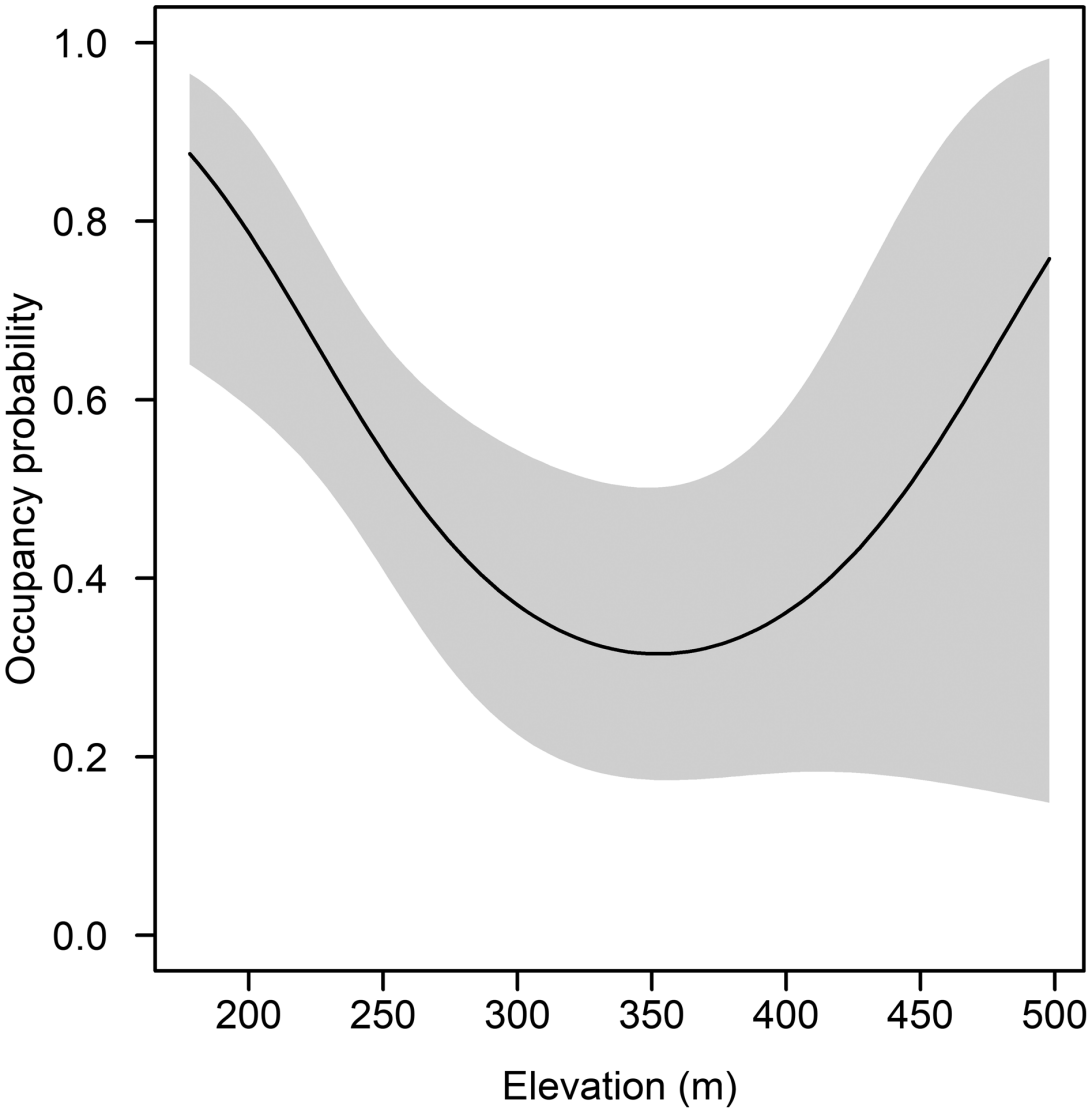
Predicted occupancy probability with 95% CIs (shaded area) for nine-banded armadillos (*Dasypus novemcinctus*) in southeastern Oklahoma, USA, during August–September (2021) as a function of a quadratic effect in elevation.

For both species, we did not find evidence for lack of fit based on the *χ*^2^ statistics (*P*_wild pigs_ = 0.183; *P*_armadillos_ = 0.454). That is, we did not find evidence of violations of model assumptions for either species. Importantly, we acknowledge that the closure assumption was likely violated for wild pigs due to their high movement capacities; however, results of our GOF test indicate that movements were likely random and occupancy results may be correctly interpreted as “use” (Gould et al. 2019). The factors associated with detection and space use varied between wild pigs and armadillos. For wild pigs, the most-supported model indicated that detection was significantly different between months (*β* = −1.56 ± 0.48 SE, 85% CI = −2.25, −0.86), and this temporal structure was present in all competing models (Table 3). When comparing months, wild pigs were >4× more likely to be detected during the second month. The most-supported model for wild pig space use included the proportion of riparian cover, but the effect was not different from zero (*β* = 5.35 ± 4.83 SE, 85% CI = −1.60, 12.31); however, the variable weight (0.68) suggested that riparian cover was important in explaining variation in wild pig space use. For armadillos, the most-supported model structure for detection included positive effects of day of year (i.e., linear trend; *β* = 0.13 ± 0.07 SE, 85% CI = 0.03, 0.22) and precipitation (*β* = 3.86 ± 1.75 SE, 85% CI = 1.34, 6.38; Table 3), while variation in occupancy was best explained by a quadratic effect in elevation (*β*_Elevation_ = −0.06 ± 0.03 SE, 85% CI = −0.10, −0.02; *β*_Elevation_^2^ < 0.01 ± < 0.01 SE, 85% CI = < 0.01, 0.01; Table 3) with armadillo occupancy being lowest at moderate elevations (Fig. 2). Mean camera site elevation differed between San Bois (mean = 309 m, range: 163 to 515 m) and James Collins (mean = 248 m, range: 181 to 394 m); however, a study area covariate received no support in explaining additional variation in armadillo occupancy, indicating that differences in elevational distribution were unimportant. Variable importance weights demonstrated high importance of the quadratic trend in elevation (0.70) and moderate importance for day of year (0.48) and precipitation (0.60).

**Table 3.** Model selection results for single-species occupancy (ψ) and detection (*p*) of wild pigs (*Sus scrofa*) and nine-banded armadillos (*Dasypus novemcinctus*) in southeastern Oklahoma, USA, during August to September (2021), with models ranked by Akaike’s information criterion adjusted for small samples (AIC_*c*_) and differences in AIC_*c*_ (ΔAIC_*c*_, where Δ_*i*_ = AIC_*ci*_ − AIC_*cmin*_) reported with number of parameters (*K*), Akaike weight (*w*_*i*_), and deviance (Dev.). Only models within 2 ΔAIC_*c*_ with a *w*_*i*_ ≥ 0.02 and the null (intercept only) model (for comparison) were presented.

Model structure^a^	*K*	AIC_*c*_	ΔAIC_*c*_	*w*_*i*_	Dev.
*Wild Pig*					
ψ(Riparian), *p*(*T*_*month*_)	4	231.18	0.00	0.04	222.69
ψ(Riparian), *p*(*T*_*month*_ + Temp)	5	231.48	0.30	0.03	220.74
ψ(Riparian + Deciduous), *p*(*T*_*month*_)	5	231.80	0.62	0.03	221.06
ψ(.), *p*(*T*_*month*_)	3	231.99	0.81	0.02	225.70
ψ(Riparian + Deciduous), *p*(*T*_*month*_ + Temp)	6	232.17	0.99	0.02	219.12
ψ(Stream + Riparian), *p*(*T*_*month*_)	5	232.20	1.02	0.02	221.46
ψ(.), *p*(*T*_*month*_ + Temp)	4	232.25	1.07	0.02	223.76
ψ(Riparian + Shrub), *p*(*T*_*month*_)	5	232.30	1.12	0.02	221.56
ψ(Stream + Riparian), *p*(*T*_*month*_ + Temp)	6	232.55	1.37	0.02	219.50
ψ(Riparian + Elevation), *p*(*T*_*month*_)	5	232.63	1.45	0.02	221.89
ψ(Riparian + Shrub), *p*(*T*_*month*_ + Temp)	6	232.64	1.46	0.02	219.59
ψ(Riparian + Deciduous + Elevation), *p*(*T*_*month*_)	6	232.73	1.55	0.02	219.68
ψ(Stream + Riparian + Deciduous), *p*(*T*_*month*_)	6	232.83	1.65	0.02	219.78
ψ(Shrub), *p*(*T*_*month*_)	4	232.83	1.65	0.02	224.34
ψ(.), *p*(.)	2	243.02	11.84	<0.01	238.88
*Nine-banded Armadillo*					
ψ(Elevation^2^), *p*(*T* + Precip)	6	605.71	0.00	0.04	592.66
ψ(Riparian + Elevation^2^), *p*(*T* + Precip)	7	606.22	0.51	0.03	590.80
ψ(Elevation^2^), *p*(.)	4	606.33	0.62	0.03	597.84
ψ(Riparian + Elevation^2^), *p*(.)	5	606.73	1.01	0.02	595.99
ψ(Riparian), *p*(*T* + Precip)	5	606.92	1.20	0.02	596.18
ψ(Elevation^2^), *p*(Precip)	5	607.18	1.47	0.02	596.44
ψ(Riparian + Deciduous), *p*(*T* + Precip)	6	607.39	1.68	0.02	594.34
ψ(Shrub + Elevation^2^), *p*(*T* + Precip)	7	607.55	1.84	0.02	592.14
ψ(Elevation^2^), *p*(*T *+ Temp + Precip)	7	607.59	1.88	0.02	592.18
ψ(Riparian), *p*(.)	3	607.62	1.91	0.02	601.33
ψ(Riparian + Elevation^2^), *p*(Precip)	6	607.63	1.92	0.02	594.58
ψ(.), *p*(.)	2	611.12	5.41	<0.01	606.98

^a^Covariates: *T* = linear trend in detection; *T*_*month*_ = detection differs between months; Temp = mean daily minimum temperature (°C); Precip = mean daily precipitation (mm); Land-cover types (i.e., Riparian, Deciduous, and Shrub) = proportion of each land-cover type within a 200-m buffer; Stream = distance (m) to nearest classified stream; Elevation = elevation (m) of site; Elevation^2^ = quadratic effect of elevation; Null (intercept only) models are indicated with a “.”.

### Co-occurrence

**Fig. 3.**
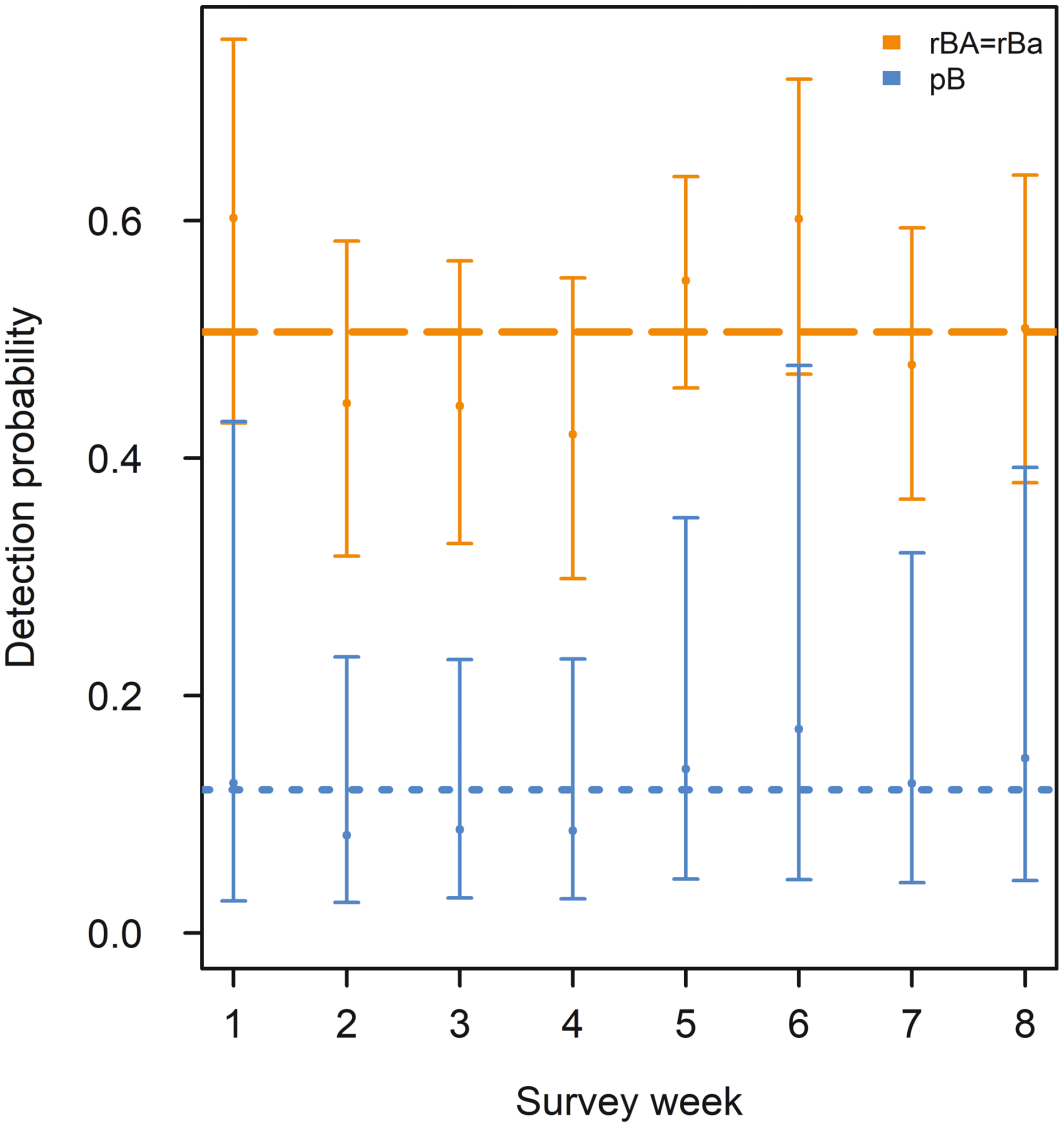
Estimated weekly probability of detection with 95% CIs for nine-banded armadillos (*Dasypus novemcinctus*) in the absence (*p*^B^) and presence (*r*^BA^ = *r*^Ba^) of wild pigs (*Sus scrofa*) during August to September (2021) in Oklahoma, USA. Overall mean detection of armadillos throughout the sampling season was significantly greater at sites used by wild pigs (0.51) than those not used by wild pigs (0.12).

Initial co-occurrence models including environmental covariates for occupancy from the most-supported single-species models introduced substantial convergence issues (i.e., only 5 [41%] models converged). Our primary focus of co-occurrence modeling was to evaluate the potential influence of wild pigs, and this could be accomplished without inclusion of covariates on occupancy. Therefore, we excluded environmental covariates on occupancy within the co-occurrence framework but retained detection covariates to account for imperfect detection. After removing occupancy covariates, all co-occurrence models converged. The most-supported co-occurrence model suggested that armadillo occupancy was not influenced by wild pig occurrence (i.e., ψ^BA^ = ψ^Ba^), a structure consistent in the second most-supported model (i.e., the only other model within 2 AIC_*c*_; Table 4); inspecting the third and fourth most-supported models indicated that ψ^BA^ ≠ ψ^Ba^ introduced a pretending variable on each of the 2 most-supported models (Arnold 2010). The most-supported co-occurrence model suggested that wild pig detection was similar in the presence or absence of armadillos (i.e., *p*^A^ = *r*^A^), and this pattern was supported across the model set (i.e., for all models with Akaike weights > 0, *p*^A^ = *r*^A^ was always more supported when compared to the model that differed only by *p*^A^ ≠ *r*^A^; Table 4). In contrast, co-occurrence models indicated that while accounting for environmental and temporal effects, armadillo detection was influenced further by the occurrence of wild pigs but not by the detection of wild pigs during the same survey (Table 4; Fig. 3). The armadillo detection structure was identical in each of the 4 top models in our model set, which collectively accounted for ~92% of the model weight (Table 4) and indicated that the observed pattern was a strong factor driving model selection results. Hence, our results suggested that armadillo detection, but not occupancy, was associated with wild pig space use. Indeed, model-averaged detection estimates showed that armadillo detection was 3.5 to 5.1× higher when wild pigs were present, regardless of wild pig detections during a survey period (Fig. 3).

**Table 4.** Model selection results for conditional 2-species occupancy and detection models of wild pigs (*Sus scrofa*; pigs) and nine-banded armadillos (*Dasypus novemcinctus*; armadillos) in southeastern Oklahoma, USA, during August to September (2021), with models ranked by Akaike’s information criterion with small sample size correction (AIC_*c*_) and differences in AIC_*c*_ (ΔAIC_*c*_, where Δ_*i*_ = AIC_*ci*_ − AIC_*cmin*_) and reported with number of parameters (*K*), Akaike weight (*w*_*i*_), and deviance (Dev.). Models of occupancy for pigs and armadillos excluded environmental predictors, whereas models for detection included predictors in the most-supported species-specific detection models (i.e., pig detection ~ monthly temporal trend; armadillo detection ~ linear time trend + mean daily precipitation).

Model Structure^a^	*K*	AIC_*c*_	ΔAIC_*c*_	*w*_*i*_	Dev.
ψ^A^, ψ^BA^=ψ^B^a, *p*^A^ = *r*^A^, *p*^B^, *r*^BA^ = *r*^B^a	10	828.46	0.00	0.43	805.56
ψ^A^, ψ^BA^=ψ^B^a, *p*^A^, *r*^A^, *p*^B^, *r*^BA^ = *r*^B^a	12	829.40	0.94	0.27	801.18
ψ^A^, ψ^BA^, ψ^B^a, *p*^A^ = *r*^A^, *p*^B^, *r*^BA^ = *r*^B^a	11	830.84	2.39	0.13	805.32
ψ^A^, ψ^BA^, ψ^B^a, *p*^A^, *r*^A^, *p*^B^, *r*^BA^ = *r*^B^a	13	831.65	3.20	0.09	800.67
ψ^A^, ψ^BA^=ψ^B^a, *p*^A^ = *r*^A^, *p*^B^, *r*^BA^, *r*^B^a	13	833.23	4.77	0.04	802.24
ψ^A^, ψ^BA^=ψ^B^a, *p*^A^, *r*^A^, *p*^B^, *r*^BA^, *r*^B^a	15	834.74	6.28	0.02	797.98
ψ^A^, ψ^BA^, ψ^B^a, *p*^A^ = *r*^A^, *p*^B^, *r*^BA^, *r*^B^a	14	835.79	7.34	0.01	801.96
ψ^A^, ψ^BA^, ψ^B^a, *p*^A^, *r*^A^, *p*^B^, *r*^BA^, *r*^B^a	16	837.17	8.72	0.01	797.40
ψ^A^, ψ^BA^=ψ^B^a, *p*^A^ = *r*^A^, *p*^B^ = *r*^BA^ = *r*^B^a	7	842.95	14.50	0.00	827.53
ψ^A^, ψ^BA^, ψ^B^a, *p*^A^ = *r*^A^, *p*^B^ = *r*^BA^ = *r*^B^a	8	843.51	15.06	0.00	825.67
ψ^A^, ψ^BA^, ψ^B^a, *p*^A^, *r*^A^, *p*^B^ = *r*^BA^ = *r*^B^a	10	844.08	15.62	0.00	821.18
ψ^A^, ψ^BA^=ψ^B^a, *p*^A^, *r*^A^, *p*^B^ = *r*^BA^ = *r*^B^a	9	847.47	19.02	0.00	827.13

^a^Model abbreviations: ψ^A^ = occupancy of pigs; ψ^B^ = occupancy of armadillos in the presence (ψ^BA^) and absence (ψ^Ba^) of pigs; *p*^A^ = detection of pigs in the absence of armadillos; *r*^A^ = detection of pigs in the presence of armadillos; *p*^B^ = detection of armadillos in the absence of pigs; *r*B = detection of armadillos when pigs were present and either detected (*r*^BA^) or not detected (*r*^Ba^).

### Temporal activity.

**Fig. 4.**
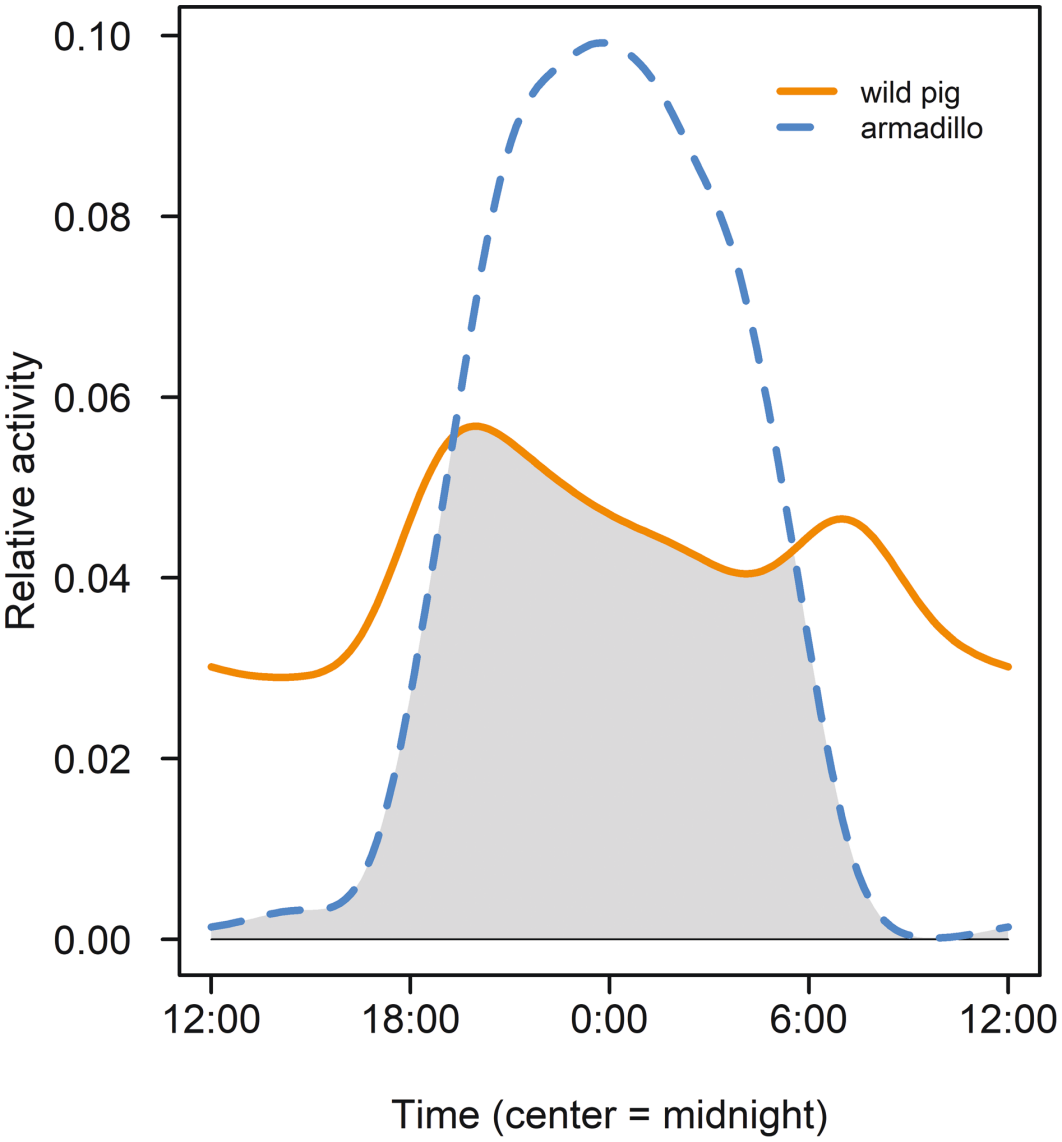
Diel activity overlap between wild pigs (*Sus scrofa*) and nine-banded armadillos (*Dasypus novemcinctus*) during August to September (2021) in Oklahoma, USA, using kernel density plots derived from image capture times with 30-min buffers within a 24-h period; overlap in activity (∆ _4_ = 0.63) is indicated by the shaded area.

Diel activity patterns indicated a lack of temporal segregation between wild pigs and armadillos. Within a 24-h cycle, wild pigs and armadillos exhibited a moderate-to-high coefficient of overlapping activity (∆ _4_ = 0.63, 95% CI = 0.58, 0.69; Fig. 4). The Watson Two-Sample Test for Homogeneity indicated that species activity distributions were not equal (*U*^2^_0.05_ = 3.45, *P* < 0.001), suggesting that species-specific activity distributions significantly differed. Neither armadillo (*U*^2^_0.19, 0.05_ = 14.67, *P* < 0.001) nor wild pig activity (*U*^2^ = _0.19, 0.05_ = 0.31, *P* < 0.05) were uniformly distributed. Therefore, we rejected the null hypothesis that data were uniformly distributed around a circle, suggesting that activity patterns were concentrated around central means.

## Discussion

Understanding the relative effects of factors influencing space-use patterns of a species can elucidate ecological or community processes. We investigated co-occurrence patterns of wild pigs (a large-bodied invasive species) and armadillos (a smaller native species), which have both expanded their range across North America (Bevins et al. 2014; Loughry et al. 2015), to evaluate the potential for wild pigs to influence space use patterns of armadillos. Although we predicted that armadillo space use would be positively associated with wild pig space use (Saldo et al. 2023), we did not observe an effect of wild pig occurrence on armadillo space use during our study. Consistent with our predictions, however, armadillo detection (or site-use intensity) dramatically increased in response to wild pig presence, elucidating subtle interspecific interactions that would otherwise be masked by course-scale spatial analyses (e.g., joint species distribution modeling; Pollock et al. 2014).

Despite similar foraging behaviors (e.g., use of keen olfactory senses and rooting; McBee and Baker 1982; Barrios-Garcia and Ballari 2012) and sympatry across the southeastern United States, interspecific interactions of wild pigs and armadillos have received little attention. In South Carolina, armadillos have been documented regularly using wild pig wallows (Eckert et al. 2019) and using sites with greater wild pig activity during spring, but there was no effect of wild pigs on armadillos in other seasons (Saldo et al. 2023). Both armadillos and wild pigs exploit soil invertebrates (Sikes et al. 1990; Marshall et al. 2020), and when soil invertebrate resources are high or resilient (e.g., in spring when soil moisture is high; Davis et al. 2006), higher armadillo intensity of use in areas with wild pigs may reflect resource matching (e.g., Ranta and Kaitala 2000; Lonsinger et al. 2017). If resource matching occurs and is accomplished via armadillos using olfactory senses to detect areas used by wild pigs, wild pigs may indirectly improve armadillo foraging efficiency. We did not observe an effect of wild pigs (positive or negative) on armadillo occupancy during our late summer (August to September) sampling season, aligning with patterns reported by Saldo et al. (2023). Additionally, our co-occurrence results (Table 3) highlight consistent detection patterns for armadillo in the 4 top-ranked models.

The potential negative effects of wild pigs on armadillos are also not well understood. Although armadillos have been detected in a dietary analysis of wild pigs (Cervo and Guadagnin 2020), it could not be determined if armadillos were killed or scavenged, and physical (i.e., dense protective scales) and behavioral adaptations of armadillos limit their susceptibility to predation (McBee and Baker 1982). Consequently, we did not expect predation by wild pigs to negatively influence armadillo space use. Habitat degradation caused by the rooting and pugging (i.e., disturbance common in moist soils in which foot prints compact soil and remain long after the soil dries; Marshall et al. 2020) by wild pigs can lead to significant declines in soil moisture availability and associated invertebrate communities (Barrios-Garcia and Ballari 2012; Marshall et al. 2020), and this may be exacerbated by wild pig consumption of soil invertebrates (i.e., exploitative competition) during dry seasons. The ecological impacts imposed by wild pigs may, consequently, explain the patterns that we observed in armadillo behavior. While we did not detect a negative influence of wild pigs on armadillo occupancy, we observed an increase in armadillo detection (site-use intensity) at sites used by wild pigs, which could be related to several factors. Armadillos rely on burrows and have small home ranges (McBee and Baker 1982; Gammons et al. 2009b) and short movement distances (McDonough and Loughry 1997), which may limit their ability to shift core activity areas. If wild pigs substantially decrease soil invertebrate abundance (either directly or indirectly), armadillos may need to alter their foraging activity by increasing foraging time or distance traveled between resource patches (i.e., optimal foraging theory; Charnov 1976; Stephens and Krebs 1986). Thus, the increase in armadillo detection (intensity of use) that we observed at sites with wild pigs may be a consequence of altered foraging behavior (Humphrey 1974; McDonough and Loughry 1997; Taulman and Robbins 2014).

Our single-species results supported patterns observed in other systems. Wild pig detection increased >4-fold in the second month of the sampling season, which may be explained by increased availability of important diet items such as oak mast (Wood and Roark 1980; Fay et al. 2023). Mast availability usually increases in September (second survey month) at our sites and is an important food source for which wild pigs compete with native species (Fay et al. 2023). During the second month of our study, wild pigs may have increased foraging movements to capitalize on these pulse resources, leading to our observed increase in detection rates. Further, we observed patterns of space use consistent with previous research indicating that riparian cover was important, presumably due to the availability of dense cover, soil moisture, prey, and wallowing opportunities (Gaston et al. 2008; Franckowiak et al. 2018; Kramer 2021). The positive effect of riparian cover that we detected was weak though (i.e., 85% CI coverage was primarily positive, but overlapped zero) and may relate to the riparian cover being a proxy for another unmeasured predictor (e.g., thermal cover or prey), but additional data would be needed to further resolve this relationship (Sutherland et al. 2023).

Similar to wild pigs, armadillo detection increased through the sampling season. Considering the relationship between armadillo detection rates and wild pig presence (i.e., positive), wild pigs may have triggered increased armadillo site-use intensity, and therefore detection. Alternatively, the linear increase in armadillo detection through the study coincides with armadillo juvenile dispersal (Feldhamer et al. 2020), which could have increased site-use intensity via movement or local abundance; however, disentangling these factors would require further study including direct measurement of these components. Detection of armadillo also increased in response to precipitation; however, the ultimate mechanism is likely that armadillos increase foraging to exploit increased prey resource availability associated with precipitation events. Earthworms typically increase activity in soil strata with higher moisture content (i.e., near surface; reviewed in Lavelle 1988) making them more vulnerable to olfactory detection and depredation by armadillos. Armadillo occupancy was highest at low (<200 m) elevations, lower at moderate elevations, but inconclusive at the highest elevations. The higher occupancy at lower elevations may be related to prey availability (see above) or denning conditions because armadillos tend to disproportionately locate their dens in habitats that occur at lower elevations (e.g., bottomland and ephemeral riparian habitats; Zimmerman 1990) and shift to higher elevations only temporarily during periods of heavy precipitation (McBee and Baker 1982).

Many species adjust their diel activity patterns to minimize risks (e.g., predation), avoid unfavorable environmental conditions (e.g., temperature; Maccarini et al. 2015), or temporally partition resources, and wild pigs and armadillos exhibit flexibility in their diel activity patterns. Wild pigs are often nocturnal (Brivio et al. 2017) but may shift their levels of nocturnality in response to human activity (Burton et al. 2024) or exhibit diurnal activity (Stolle et al. 2015). Armadillos are also often nocturnal or crepuscular but display flexibility and can be diurnal under some conditions (McDonough and Loughry 1997; Green et al. 2016; DeGregorio et al. 2023). Armadillos in our study system were strictly nocturnal, aligning with patterns observed by 1 study in the southeastern United States (Saldo et al. 2023). Although we observed diel activity patterns for wild pigs that peaked during the crepuscular period, wild pig activity was more cathemeral than patterns observed by Saldo et al. (2023), with relatively high activity during diurnal periods. Armadillo activity in our system was highest (and unimodal) between wild pig crepuscular activity peaks, suggesting that fine-scale temporal partitioning may have occurred. Further temporal niche partitioning between these species may have occurred on a coarser (i.e., days) scale that we did not investigate.

We investigated the influence of wild pigs on armadillos using a modeling framework (i.e., co-occurrence) that assumes occurrence of a dominant species can influence the occurrence of a subordinate species, but the opposite is not true (Richmond et al. 2010). Due to the complexity of our modeling framework, we were unable to include covariates explaining occupancy (i.e., riparian cover for wild pigs and elevation for armadillos) when investigating co-occurrence patterns, which may have limited our inferences. A challenge of co-occurrence modeling is that the scale of a site (i.e., the sampling unit) is the same for both species. Consequently, when considering 2 species exhibiting different movement capacities and spatial ecologies (e.g., disparate home range sizes, group sizes), selecting an appropriately sized site can be difficult. We selected a scale best aligned with armadillos—the smaller, less mobile, and more solitary species. Therefore, the closure assumption for wild pigs may have been violated, although we observed no statistical evidence for this. Nonetheless, closure violations tend to create an upward bias in occupancy (or use; Rota et al. 2009), making it more difficult to identify existing patterns of aggregation or avoidance between species (Lonsinger 2022). Spatial co-occurrence patterns may be related to factors not explored in this study, such as resource availability, forage quality, or fine-scale temporal relationships between species. Thus, alternative analysis frameworks that couple behavioral information with fine-scale temporal relationships of spatial co-occurrence—e.g., time-to-event analyses (Watabe et al. 2022; Williams et al. 2022) or analyses incorporating continuous-time detection processes (Kellner et al. 2022)—may help disentangle the behavioral mechanisms underlying spatial co-occurrence between these 2 species but would require a more robust dataset than was available.

### Implications

Mechanisms driving the range of a species are complex (Sexton et al. 2009), and mammal communities are broadly structured along temperature and precipitation gradients (Kays et al. 2024). However, there is growing evidence that interspecific interactions can facilitate or limit species-specific range expansions (e.g., via assembly rules or priority effects; Peng and Zhou 2014; Svenning et al. 2014). Understanding interspecific interactions and their influence on species range expansions relative to environmental conditions is complex and highlights the need for large-scale and long-term research employing multiseason (i.e., dynamic) models capable of formally considering interacting species (Svenning et al. 2014; Fidino et al. 2019; Zhao et al. 2022). While we observed nuanced evidence of interspecific interactions between 2 co-expanding species, we emphasize that our findings could be context-dependent. The fine-scale patterns observed in Oklahoma may reflect interspecific interactions in other areas of sympatry, but we caution that interspecific interactions can vary across systems and community assemblages and might differ in areas of longer sympatry versus areas of expansion.

## Data Availability

Data available upon request.
